# A Culinary Misadventure: A Case Report of Shiitake Dermatitis

**DOI:** 10.21980/J8X936

**Published:** 2021-10-15

**Authors:** Chia-Yuan Michael Lee, Michael T Dalley

**Affiliations:** * Mount Sinai Medical Center—Miami Beach, Department of Emergency Medicine, Miami Beach, FL

## Abstract

Shiitake dermatitis is a rare cutaneous reaction that can occur after consumption of shiitake mushrooms. It is characterized by highly pruritic lesions with a distinct whip lash appearance. Management of this condition includes reassurance and providing symptomatic relief. Although most reported cases have occurred in Asia, this rash has made appearances in the Western world. Here, we report a case of a 23-year-old female in the United States who experienced this itchy ailment. A diagnosis was made based on the presence of the recognizable lesions and dietary history. She was discharged with advice to continue symptomatic care and given reassurance about her condition. Her symptoms resolved within a few weeks. As the use of shiitake mushrooms become more widespread, it is important for clinicians to be familiar with this rash and remember to take a detailed dietary history.

**Topics:**

Rash, dermatology, toxicology, shiitake mushrooms.

**Figure:**
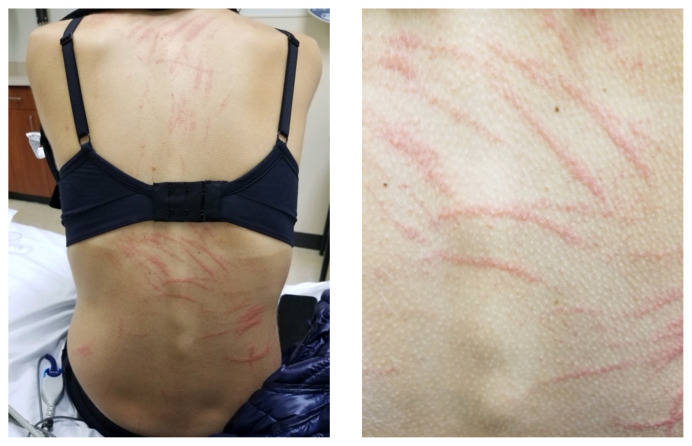


## Brief introduction

Shiitake mushrooms (*Lentinula edodes)* have long been a staple of Asian cuisine and medicine. Coveted for its umami flavor and meaty texture, these mushrooms also confer potential therapeutic benefits against illnesses such as hypertension, hyperlipidemia, and cancer.^1^ With the growth in popularity of Asian cuisine and globalization of cultures, the international use of this delicacy has become so ubiquitous that it is now the second most cultivated edible mushroom worldwide.^2^,^3^ Today, the mushroom is available fresh, dried, canned and as part of seasonings, supplements, sauces, drinks, and candy.^3^

In rare instances, consumption of shiitake mushrooms has been associated with a dermatitis accentuated by linear erythema and considerable pruritus. The presentation of this striking rash is uncommon with only an estimated 100 reported incidents since its first reported observation.^4^ Most episodes occurred in Asia, and although there have now been several cases in Europe and the Americas, it is still a relatively extraordinary occurrence in Western countries and the United States.^5^,^6^ Below, we present a case of a young female presenting to the emergency department with shiitake dermatitis.

## Presenting concerns and clinical findings

A 23-year-old female without any past medical history or family history presented to the emergency department with a two-day history of an intensely pruritic rash on her back. When the breakout first started, she only noted a few isolated itchy lesions on her upper back. She went to an urgent care where she received diphenhydramine, methylprednisolone, and a hydrocortisone-clotrimazole cream. Despite using these medications, the lesions became more widespread, and her itching intensified. Because she was breastfeeding, she presented to the emergency department with concerns that whatever was causing her rash would affect her breastmilk. She denied fever, gastrointestinal symptoms, neurological symptoms, joint swelling, body aches, throat irritation or swelling, and shortness of breath. Her vitals revealed a blood pressure of 102/71, heart rate of 67 beats per minute, respiratory rate of 18 breaths per minute, a temperature of 37.3 degrees Celsius, and an oxygen saturation of 99% on room air. Her physical exam revealed multiple streaked lesions on her back.

## Significant findings

Close visual examination revealed erythematous linear papules on her upper and lower back. No bullae, drainage, or sloughing of the skin was present. The rest of her body, including palms, soles, and mucosa, was spared.

## Patient course

When it comes to rashes, every clinician, especially those in the emergency department, needs to consider etiologies that can be life-threatening. For this patient, a number of potentially dangerous cutaneous manifestations were considered, but her presentation was not consistent with them. Stevens-Johnson Syndrome and toxic epidermal necrolysis were unlikely given that she was not on any medications, had no prodromal symptoms, and the lesions did not slough nor affect her oral mucosa. The lack of medication use or exposure to chemotherapy made a potential drug reaction less likely. She had no fever, altered mental status, purpuric lesions, or neck rigidity that would suggest meningitis. Her lesions did not blister nor did they have an appearance consistent with lesions seen in pemphigus vulgaris or bullous pemphigoid. Syphilis and HIV were unlikely since she did not have risk factors, such as drug use or high-risk exposure, and she had undergone testing for sexually transmitted diseases during her recent pregnancy. The lesions were not self-inflicted nor caused by another individual. No one else she lived with had any symptoms, and she denied any recent bug bites. She denied any new shampoo, soaps, detergents, or any obvious potential skin irritants. Her presentation was not consistent with a cellulitis. Autoimmune disease was deemed unlikely given the absence of any other symptoms and negative past medical and family history. Scombroid and ciguatera were also unlikely given that she denied eating any fish, and her rash did not have a diffuse flushed appearance.

However, upon further questioning of her food intake, the patient reported that one day prior to onset of symptoms she had eaten shiitake mushrooms. Given her dietary exposure and the discernible lesions, a diagnosis of shiitake dermatitis was made.

Despite having eaten shiitake mushrooms frequently in the past, she had never had this skin eruption before. Nevertheless, the patient was reassured that her lesions would resolve on their own and that she could continue breastfeeding without concern. She was instructed to continue using the medications advised by the urgent care for symptomatic relief.

On follow-up, she reported that the rash had resolved after approximately 3 weeks. She noted that the medications provided mild relief. Despite this unfortunate experience, she has not shied away from shiitake mushrooms. She continues to eat them regularly and has not had any recurrence of symptoms.

## Discussion

First described in 1977, shiitake dermatitis is an intensely pruritic toxicoderma that can occur after eating shiitake mushrooms.^2^ Characterized by linear, erythematous papules resembling lashes from a whip, it is described as a flagellate dermatitis - a name derived from the Flagellants, a medieval religious sect whose members would whip themselves as a demonstration of their faith.^6^,^7^ The lesions typically appear 24 to 48 hours after consumption of the mushroom^2^ and almost always involve the trunk, commonly involve the limbs, less commonly involve the face and neck, and rarely involve the hands and feet.^8^ Although the lesions may rarely present as petechiae, purpura, plaques, pustules, and even morbilliform, the notable flagellate lesions appear in nearly all cases.^3^,^8^ It is uncommon, but possible, for patients to also experience fever, diarrhea, paresthesia, involvement of oral or eye mucosa, and dysphagia.^8^,^9^

Although the exact pathophysiology is unclear, the rash is thought to be due to a toxic reaction to lentinan, a polysaccharide component of the mushroom that stimulates interleukin-1 which results in vasodilation and inflammation.^6^,^7^,^10^ In fact, a similar cutaneous eruption has been observed in a select few patients undergoing chemotherapy treatment with lentinan.^2^ Because lentinan is denaturized around 130–145 degrees Celsius, it has been suggested that only exposure to raw or undercooked mushrooms would cause the dermatitis.^2^,^3^,^6^ In one report, a patch test using mushrooms cooked at 100 degrees Celsius resulted in the skin eruption but not when the mushrooms were cooked at 150 degrees Celsius.^11^ However, there have also been multiple cases of shiitake dermatitis after the consumption of cooked mushrooms^5^,^7^ and even after drinking a beverage containing shiitake mushroom extract.^5^

There is some debate as to whether shiitake dermatitis is truly the result of a toxic reaction.^3^,^6^–^8^ A few studies have demonstrated that certain human leukocyte antigen alleles and cytokine profiles make individuals more susceptible to generating a skin reaction when exposed to antigens, and an argument could be made that a similar connection could explain shiitake dermatitis although a specific marker has yet to be identified.^6^,^8^ There is also some evidence that a T-cell reaction could also be the root of the rash.^7^,^8^

The development of the rash has been proposed to be the result of Koebnerization,^2^,^11^ a phenomenon where trauma elicits new lesions in previously unaffected areas. Whether or not the lesions can be attributed to the Koebner phenomenon is still disputable. Garg et al noted that the lesions were not reproducible by scratching.^12^ However, Ade et al. reported that a patient who scratched his initials into his flank after eating raw shiitake mushrooms developed a rash in the form of his initials 44 hours later.^11^ They hypothesized that there was a critical time period where serum lentinan levels were high and that scratching during this time would result in the most severe lesions.^11^

Recently, another theory regarding the cause of shiitake dermatitis has surfaced. It was suggested that occurrence of this phenomenon depends on the way the mushroom is produced^13^: “log-grown” where the spores are directly placed onto tree logs versus “substrate-grown” where the spores are spread onto sawdust.^6^ It was noted that there have been very few cases of shiitake dermatitis in China where the mushrooms are substrate-grown while most of the cases have been in Japan where the mushrooms are log-grown.^13^ Furthermore, there were almost no reported cases in Japan after the government made a switch to substrate-grown mushrooms.^13^

Although these whip-like lesions are uncommon, there are several other possible causes. Because the term “flagellate dermatitis” was originally coined to describe bleomycin-induced dermatitis, bleomycin exposure must be considered.^14^ Both bleomycin-induced dermatitis and shiitake dermatitis have similar non-specific histological features: epidermal spongiosis, perivascular infiltrate with lymphocytes and eosinophils, and keratinocyte decay.^7^,^9^ Additionally, bleomycin is a sulfur-containing peptide that is structurally comparable to a sulfur element of the shiitake mushroom.^9^,^15^ Unlike shiitake dermatitis however, bleomycin-induced dermatitis typically results in hyperpigmentation of the skin after the initial lesions resolve.^5^–^7^ Differential diagnoses also include adult-onset Still’s disease, jellyfish stings, and actual whip-lashes.^15^ It is fairly easy to rule out bleomycin-induced dermatitis, jellyfish stings, and whip lashes from simply taking a history, while adult-onset Still’s disease should manifest other symptoms such as joint pains and fever. Overall, diagnosis of shiitake dermatitis is dependent on recognition of the flagellate lesions and confirming dietary exposure. Histological studies and blood work are non-specific, and provocative testing, such as skin prick and patch tests, are unreliable.^2^,^3^,^6^

Although patients may be irritated by the itching and alarmed by the appearance of the rash, the condition is self-limiting and typically resolves in a matter of days to weeks.^2^,^5^–^10^ Antihistamines and topical steroids may be used in an attempt to provide symptomatic relief although it is debatable if these interventions affect the duration or severity of symptoms.^3^,^6^ In fact, almost 30% of patients reported no relief with treatment.^8^ Systemic steroids appear to speed up resolution of symptoms by about three days; however, this data lacks power and was gathered from a review of cases and not from a randomized controlled trial.^8^ Sunlight has been noted to exacerbate the symptoms in a few cases so avoidance of sun exposure may be suggested.^9^ One may also advise avoidance of shiitake mushrooms or at least recommend that they are thoroughly cooked prior to ingestion. In any event, the patient should be re-assured that the lesions will likely resolve on their own in time.

For clinicians who are unfamiliar with this condition, the diagnosis can be difficult to make and delayed. In one extreme case, a patient suffered from the rash intermittently for 16 years before finally receiving a diagnosis.^12^ Dermatologists may be more likely to be cognizant of shiitake dermatitis, but many emergency physicians might not have adequate experience with this condition because dermatological complaints only comprise approximately 3% of ED visits.^16^ Although only 2% of those complaints are triaged as emergent, many of them, like shiitake dermatitis, can be worrisome to patients.^16^ Recognition of this cutaneous reaction will allow physicians to allay the fears of patients and provide them with proper instructions. As shiitake mushrooms become more commonplace in Western diets, it would be prudent to be aware of the enigma of shiitake mushroom-induced flagellate dermatitis.

## Supplementary Information




