# Supplementary figures and images for: A Culinary Misadventure: A Case Report of Shiitake Dermatitis
Source: J Educ Teach Emerg Med. 2021 Oct 15;6(4):V15–8. doi: 10.21980/J8X936 (PMC10332740; doi:10.21980/J8X936)

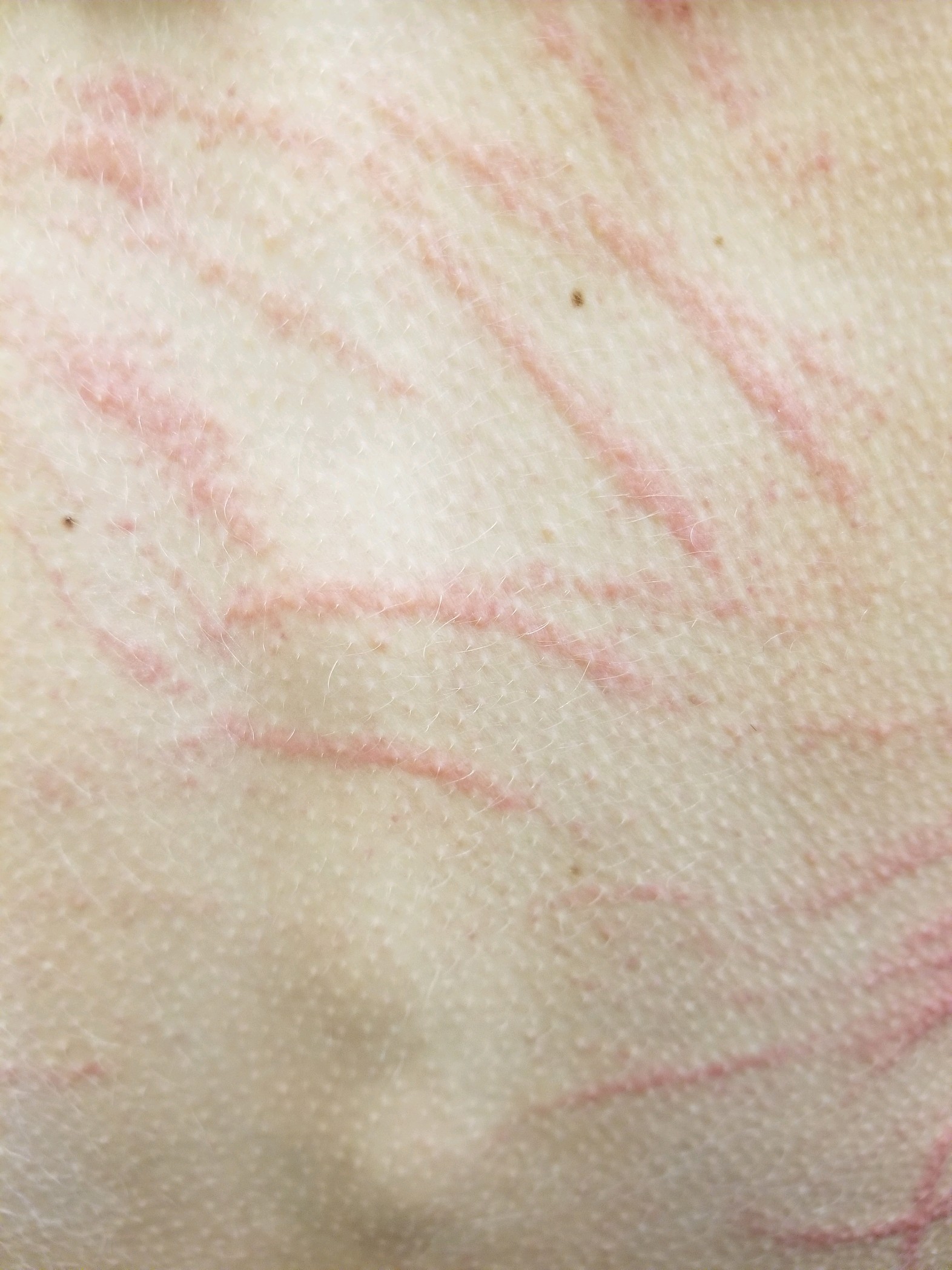

Supplement: Supplementary file 1 [file JETem-6-4-V15-supp1.jpg]

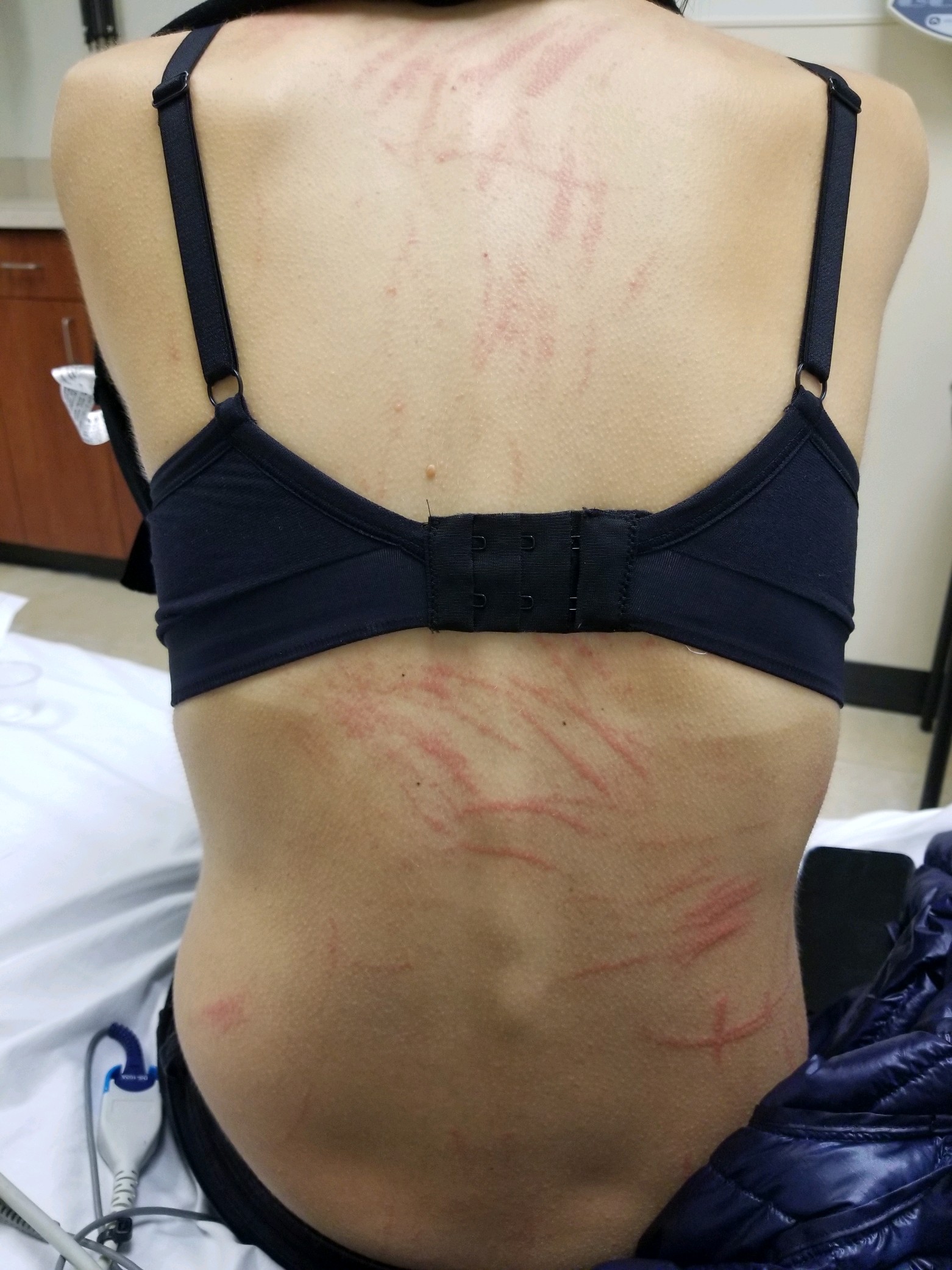

Supplement: Supplementary file 2 [file JETem-6-4-V15-supp2.jpg]
